# Commentary: Establishing zebrafish as a model to study the anxiolytic effects of scopolamine

**DOI:** 10.3389/fphar.2018.00293

**Published:** 2018-04-03

**Authors:** Murilo S. de Abreu, Ashton J. Friend, Tamara G. Amstislavskaya, Allan V. Kalueff

**Affiliations:** ^1^Bioscience Institute, University of Passo Fundo, Passo Fundo, Brazil; ^2^Postgraduate Program in Pharmacology, Federal University of Santa Maria, Santa Maria, Brazil; ^3^Neuroscience Program, School of Science and Engineering, Tulane University, New Orleans, LA, United States; ^4^The International Zebrafish Neuroscience Research Consortium, Slidell, LA, United States; ^5^Research Institute of Physiology and Basic Medicine, Novosibirsk, Russia; ^6^School of Pharmacy, Southwest University, Chongqing, China; ^7^Institute of Translational Biomedicine, St. Petersburg State University, St. Petersburg, Russia; ^8^Institute of Experimental Medicine, Almazov National Medical Research Center, St. Petersburg, Russia; ^9^Ural Federal University, Ekaterinburg, Russia; ^10^Russian National Granov's Research Center for Radiology and Surgical Technologies, Pesochny, Russia; ^11^Laboratory of Translational Biopsychiatry, Research Institute of Physiology and Basic Medicine, Novosibirsk, Russia; ^12^ZENEREI Research Center, Slidell, LA, United States

**Keywords:** scopolamine, Zebrafish, anxiety, hallucinogens, animal models

Combatting human brain disorders and searching for novel personalized therapies are becoming major, unmet medical problems (Henney, 2012). Therefore, screening for novel CNS compounds is critically important for translational biomedicine (Hurko and Ryan, 2005; Denayer et al., 2014). Important strategies in this field include (1) developing novel, more valid neurobehavioral tests, and paradigms (Echevarria et al., 2017), (2) creation of high-throughput screening platforms (Filip et al., 2012), and (3) widening the range of model organisms (Rine, 2014). Zebrafish *(Danio rerio*) are rapidly emerging as powerful, high-throughput vertebrate models for CNS drug discovery (Kalueff et al., 2014a,b; Khan et al., 2017). A recent study establishing zebrafish model for studying psychotropic effects of scopolamine is an interesting, encouraging development in this direction (Hamilton et al., 2017).

Scopolamine is a well-studied muscarinic cholinergic antagonist, with multiple effects in the peripheral and central nervous systems, and various side-effects. The drug has a long history of use in biomedicine to treat a wide range of conditions, including motion sickness (Schmäl, 2013; Golding and Gresty, 2015) and recovery from anesthesia (Kolodzie and Apfel, 2009; Norton et al., 2011). However, its exact psychopharmacological profile remains complex and poorly understood (Fredrickson et al., 2008). As recent clinical evidence shows that scopolamine is also effective at reducing symptoms of depression and anxiety, the reported anxiolytic-like effects of this drug in zebrafish seem quite possible (Hamilton et al., 2017). Specifically, scopolamine was anxiolytic in zebrafish tested in the novel approach and the novel tank diving tests, and, reaching maximum effect at a 800 μM dose, also decreased zebrafish shoaling (an effect seemingly opposite to expected pro-shoaling response to stress and/or anxiogenic stimuli) (Hamilton et al., 2017). In contrast to this profile, however, rats and mice treated with scopolamine consistently display increased anxiety in standard behavioral tests, thus contradicting cited human studies and presented zebrafish findings (Hamilton et al., 2017). Furthermore, not all zebrafish data support anxiolytic profile of scopolamine. For instance, an earlier study in adult zebrafish (Cho et al., 2012) reported no anxiolytic effects for scopolamine, also noting its ability to suppress anxiolytic-like effect of physostigmine, an acetylcholinesterase inhibitor. Contrary to Hamilton et al. (2017), but in line with rodent data mentioned above, these results suggest that zebrafish anxiety can be reduced by cholinergic signaling, and is partly mediated by muscarinic receptors (Cho et al., 2012). Taken together, these discrepancies merit further discussion. Since Scientific Reports do not publish Commentaries, these considerations will be discussed here, given their potential importance for further dissecting scopolamine CNS action *in vivo*.

Notably, in addition to modulating affective (anxiety/depression) phenotypes comprehensively evaluated in zebrafish (Hamilton et al., 2017), scopolamine also has two other well-established CNS effects—memory-impairing and hallucinogenic. For instance, scopolamine has long been known to induce cognitive deficits, consistently reported in both clinical (Atri et al., 2004; Green et al., 2005) and rodent studies (Lee et al., 2017, 2018; Skalicka-Wozniak et al., 2018). There is also rich literature on memory-impairing effects of acutely given scopolamine in zebrafish in various paradigms (Kim et al., 2010; Richetti et al., 2011; Cognato Gde et al., 2012; Bortolotto et al., 2015; Rajesh and Ilanthalir, 2016; Zanandrea et al., 2018). For example, the drug does not affect general locomotion in zebrafish, reduces T-maze novel arm exploration (Cognato Gde et al., 2012; Zanandrea et al., 2018) and opposes lithium- and nicotine-induced cognitive enhancement (Braida et al., 2014a,b; Zanandrea et al., 2018). Given the well-known memory-anxiety interplay in animal models (Kalueff and Murphy, 2007), one may consider behaviors produced by a hypothetical amnestic agent in rodent or zebrafish anxiety tests, such as especially the novel object recognition and the novel tank test (Hamilton et al., 2017). The latter is a powerful, well-established model for zebrafish behavioral neurophenotyping, particularly sensitive to anxiety manipulations (Khan et al., 2017). Since most of such models are based on novelty exposure, one of the main effects potentially observed in these paradigms from scopolamine and related amnestic compounds in any model organism in general can be reduced object recognition and poor intra-trial habituation, which may mask itself for anxiolytic-like increased “exploration” (Kalueff and Murphy, 2007). In essence, animals under scopolamine may not remember well the object or area they inspected/visited, thus requiring more additional exploration bouts in novelty tests and also displaying reduced social recognition (and, hence, cohesion) in shoaling tests. Both rodent (Akkerman et al., 2012) and zebrafish (Kyzar and Kalueff, 2016) data on scopolamine action seem to support this possibility, and, thus, it is plausible that the similar rationale (rather than anti-anxiety action *per se*) can contribute to seemingly “anxiolytic”-like effects of scopolamine reported in zebrafish (Hamilton et al., 2017).

Another well-known CNS effect of scopolamine is its hallucinogenic action (Burillo-Putze et al., 2013). The growing interest in hallucinogenic psychopharmacology (Kyzar et al., 2017) has recently been expanded to zebrafish models (Kyzar and Kalueff, 2016), thus meriting further consideration here. Can the observed behavioral effects of scopolamine (Hamilton et al., 2017) be due to its putative hallucinogenic action? Indeed, scopolamine and other anti-cholinergic drugs, such as atropine, are traditionally known as “deliriant” hallucinogens (Burillo-Putze et al., 2013) which evoke strong delusions and hyperactivation—the profile that also strikingly differs from other known classes (psychedelics, dissociatives) of hallucinogens. Clearly, this deliriant hallucinogenic action of scopolamine, if it exists in zebrafish, may not only result in increased exploratory locomotor activity in zebrafish reported (Hamilton et al., 2017), but also disrupt shoaling behavior—another observed phenotype in this study. Consistent with this notion, social behavior is disrupted by hallucinogens in many rodent studies as well (see review in Kyzar and Kalueff, 2016), most likely due to overall confusion, lethargy, and/or sensory hallucinations. Therefore, anxiolytic action is not the only reason why zebrafish shoals may become less cohesive, and multiple other factors (e.g., reduced sociality, aberrant sensory function, motor retardation, hallucinations) acting alone or jointly may affect fish group behavior in this test. Thus, given the ability of various classes of hallucinogens to nonspecifically disrupt zebrafish shoaling regardless of their effects on anxiety (Kyzar and Kalueff, 2016), this alternative possibility (rather than proposed “anxiolysis”) in underlying zebrafish shoaling-disrupting behavior under acute scopolamine seems indeed likely.

Finally, there are clear behavioral limitations of the zebrafish model which may be relevant to scopolamine action. For instance, despite the advantages of using zebrafish as a model to study anxiety-like behavior, all animal models are limited since they cannot fully recapitulate the complex repertoire of human behavior (Kalueff et al., 2014a; Stewart et al., 2014, 2015). Furthermore, it can be difficult to distinguish between various subtypes of zebrafish anxiety-like behaviors, especially since they have not yet been fully dissected in zebrafish. Likewise, proper behavioral analyses of psychotropic effects in zebrafish tests may require sophisticated 3D-based video-tracking assays (Cachat et al., 2011; Maaswinkel et al., 2013) rather than less sensitive 2D-based behavioral data. Additionally, as zebrafish demonstrate robust strain differences in their behavioral and drug responses (Maximino et al., 2013; Kalueff et al., 2014a,b; Stewart et al., 2014, 2015), this aspect may also be considered when studying complex psychopharmacology of scopolamine in adult zebrafish.

In summary, while anti-anxiety action of scopolamine in zebrafish may not be ruled out, several additional factors (e.g., amnestic and hallucinatory profile) may confound putative anti-anxiety-like action of scopolamine in this aquatic model suggested by Hamilton et al. (2017). Likewise, clinical data supporting anxiolytic action of scopolamine, discussed in Hamilton et al. (2017) seem to only partly corroborate zebrafish findings. On the one hand, since selected human studies involved chronic or repeated administration of scopolamine, and the described zebrafish experiments used acute single doses (Hamilton et al., 2017), it is problematic to make direct translational comparisons between such findings. On the other hand, acute scopolamine may cause confusion, anxiety/fear, agitation, and irritability in humans, most likely overlapping with (or being part of) its well-known pro-arousal, delirium-inducing action. In line with this, US Food and Drug Administration (FDA) lists restlessness, confusion, agitation, and hallucinations as potential side-effects of scopolamine in humans (https://www.accessdata.fda.gov/drugsatfda_docs/nda/2001/017874_S018%20&%20S027_SCOPOLAMINE_AP.pdf). Such action not only parallels numerous rodent findings on anxiogenic-like profile of scopolamine, but, given a strong similarity of zebrafish and mammalian drug targets (Kalueff et al., 2014a), may also be part of complex zebrafish behaviors induced (most likely, in a dose-dependent manner) by this agent. Comparative analyses of this drug action profile with that of atropine—another similar cholinergic agent, are also warranted. Thus, care is necessary when interpreting scopolamine data in zebrafish, and further behavioral, pharmacological as well as biomarker (e.g., endocrine, c-fos-, neurochemical) validation is needed before concluding that acute scopolamine in zebrafish is anxiolytic (or anxiogenic), and whether this organism is a valid *in-vivo* system for detecting an anxiotropic action of this drug. Such additional validation efforts, in our opinion, may include using a battery of multiple anxiety-sensitive tests (reviewed in Kalueff et al., 2014a,b), measuring cortisol stress responses in scopolamine-treated zebrafish, bidirectionally modulating their anxiety levels (induced by scopolamine) using classical “reference” anxiogenic and anxiolytic non-cholinergic compounds, and applying specific cholinergic manipulations to increase or decrease cholinergic neurotransmission in zebrafish treated with acute (and, eventually, chronic) scopolamine, as well as atropine.

## Author contributions

All authors listed have made a substantial, direct and intellectual contribution to the work, and approved it for publication.

### Conflict of interest statement

The authors declare that the research was conducted in the absence of any commercial or financial relationships that could be construed as a potential conflict of interest.
